# Healthcare preferences of chronic disease patients under China's hierarchical medical system: an empirical study of Tianjin's reform practice

**DOI:** 10.1038/s41598-024-62118-8

**Published:** 2024-05-21

**Authors:** Da Luo, Xumin Zhu, Xinyu Qiu, Jing Zhao, Xiangfei Li, Yue Du

**Affiliations:** 1 Tianjin Key Laboratory of Cerebral Vascular and Neurodegenerative Diseases, Clinical College of Neurology, Neurosurgery and Neurorehabilitation, Tianjin Medical University, Tianjin Neurosurgical Institute, Tianjin Huanhu Hospital, Tianjin, 300350 China; 2 School of Economics and Management, Tiangong University, Tianjin, 300387 China; 3 Department of Social Medicine and Health Management, School of Public Health, Tianjin Medical University, Tianjin, 300070 China; 4 Tianjin Municipal Health Commission, Tianjin, 300070 China

**Keywords:** Hierarchical medical system, Preference for healthcare, Primary healthcare, Two-way referral, Medical alliance, Diseases, Health care

## Abstract

To alleviate the contradiction in healthcare resources, the Chinese government formally established the framework of a hierarchical medical system in 2015, which contains the following brief generalities: " separate treatment of emergencies and slows, first-contact care at the primary, two-way referral, and upper and lower linkage, ". This study systematically summarizes and models the connotations of China's hierarchical medical system and a sample of 11,200 chronic disease patients in Tianjin, the largest port city in northern China, was selected for the empirical study to investigate the relationship between chronic disease patients' policy perceptions of the hierarchical medical system and their preference for healthcare. We found that under the strategy of separate treatment, improving the healthcare accessibility, drug supply, and lowering the cost of medical care would have a positive impact on increasing the preference of patients with chronic diseases to go to the primary hospitals. Under the two-way triage strategy, improving the level of physician services, referral convenience and treatment Standards have a positive impact on chronic disease patients' preference for primary care; The impact of the hierarchical medical system on the preference for healthcare differed between groups, focusing on differences in health literacy level, age and household type; The role of " upper and lower linkage " is crucial in the hierarchical medical system and it plays a part in mediating the influence of the " separate treatment of emergencies and slows" design and the "two-way referral " order on the treatment preferences of chronic disease patients. The results of the study provide a reference for the further development of a scientific and rational hierarchical medical system in the future.

## Introduction

The unbalanced allocation of healthcare resources and the underutilization of primary care facilities are the core barriers to healthcare equity in China^1^. The Hierarchical Medical System (HMS) was formally proposed in 2009 as part of a new round of health system reform to encourage patients to seek care in primary healthcare facilities rather than large hospitals^2^. Although since the beginning of the healthcare reform, full efforts have been made to develop and support primary healthcare (PHC), while strictly limiting the uncontrolled expansion of large healthcare facilities. However, the proportion of visits to primary healthcare facilities is still decreasing year on year, and large public hospitals remain overcrowded^1^. The trend of expansion and grouping of large public hospitals is noticeable, and the superior healthcare resources are continuously concentrated in large hospitals^3,4^. After years of exploration and practice, in September 2015 the Chinese government issued the " Guiding opinions on promoting the construction of hierarchical medical treatment system ", which formally established the objectives and pathways for the HMS in order to alleviate conflicts in access to healthcare^5^. This system aims to reshape the structure of China's healthcare system and provide continuous and integrated healthcare services to residents, changing their healthcare habits^6^. Even though this policy has been in place for a long time, the actual effects of the HMS are poorly understood, especially in a typical mega-city.

The preference for healthcare refers to the perception, performance and behavior of patients to seek care when they feel unwell, present symptoms of a certain disease or feel potentially at risk of illness, considering their care condition, financial situation and healthcare facilities, etc.^7^. It is a very complex psychological and social behavior, including the patient's choice of healthcare facilities, physicians, drugs and treatment methods, as well as the patient's perception of the motivation for seeking care and the expected goals and results of the visit^8^. Therefore, patients' preference and choice are crucial factors affecting the achievement of the HMS. In existing studies related to the HMS, they has focused on the capacity of primary healthcare and has conducted extensive exploration and practice. The majority of related studies are based on the Andersen Health Service Utilization Model to investigate the influence of individual factors on healthcare-seeking behaviors, including age, gender, occupation, education level, health status, income level, etc.^9,10^, but there are few studies on how resident’s perceived perceptions of HMS implementation affects patients' actual healthcare choices and even less research from the perspective of the constitutional framework.

China's urban development is in the form of a concentration of resources in the city center spreading out in all directions with quality healthcare resources are concentrated in the city center, while healthcare facilities in the suburbs are still in their infancy. As the largest port city in northern China, Tianjin has a resident population of over 13 million. Since 2015, Tianjin has become a national demonstration city in China for the development of HMS, and with continuous development and improvement over the years, it has now become one of the most typical models for the vertical integration of a region-wide healthcare system. The evaluation of the performance of HMS in Tianjin is an effective demonstration for rating the effectiveness of the HMS in China’s typical mega-city. This study attempts to investigate the impact of the design and operation of the system on residents' preference for healthcare by considering the system design of the HMS in China with Tianjin as a representative sample.

## Theoretical and hypotheses development

### Hierarchical medical system

**Figure 1:**
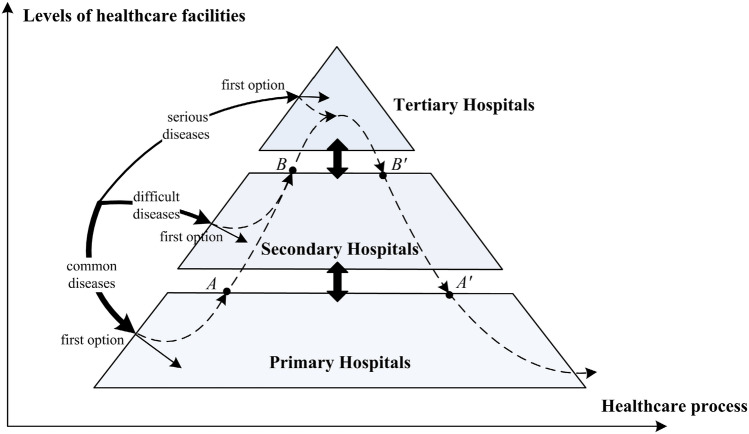
Healthcare process in the hierarchical medical system.

China's healthcare facilities are classified into three levels: primary hospitals, generally referred to community healthcare centers(CHCS) and community healthcare stations in urban areas; Secondary hospitals are generally at the county and municipal levels, while tertiary hospitals are generally at the municipal level and above^11^. In September 2015, the General Office of the State Council of China officially issued the "Guiding Opinions on Promoting the Construction of the Hierarchical Medical System ", which is an important instrument for the government to alleviate the contradiction between supply and demand of public healthcare resources. The healthcare process under the design of the HMS is shown in Fig. 1.

The basic model of HMS was defined in the form of a policy document, i.e., " separate treatment of emergencies and slows, first-contact care at the primary, two-way referral, and upper and lower linkage" which mainly include the following aspects of policy content design:

1. A well-defined healthcare system of "Separate treatment of emergencies and slows". This content is based on the disease-specific division of the various healthcare facilities’ functions, emphasizing the cooperation and division of labor within the healthcare system. According to relevant studies, diseases are classified as common, difficult or serious, where patients have different ways of seeking healthcare^12^. For common diseases, patients are more concerned with convenience and choose the nearest primary hospitals; for difficult diseases, patients may weigh between primary and more specialized secondary hospitals; for serious diseases, patients are more focused on the level of medical services and prefer the more powerful tertiary hospitals.
2. Orderly triage of "First-contact care at the primary" and "Two-way referral". This refers to the policy of encouraging patients with common and multiple diseases to seek treatment at the primary level first, based on the voluntary consent. Patients with aggravated conditions or difficult diseases are referred to high-level hospitals from the primary hospital. Patients are promoted to low-level hospitals when their disease becomes chronic or recovers. The system is intended to ensure that patients receive the appropriate healthcare services at the appropriate time and location. It promotes orderly consultation, timely diagnosis and treatment, improved treatment outcomes, and medical expense savings through optimal integration of the medical service system.
3. Tightly-knit medical alliance through "Upper and lower linkage". There are two main forms of regional medical alliance models provide joint services through cooperation: one is to use the leading hospitals in the region to take the lead in forming regional medical alliance; the other is to use municipal medical resources (tertiary hospitals) located in the region to build specialist medical alliance with regional medical resources (secondary hospitals and primary hospitals) to form partnerships^13^. Due to medical resource constraints, rural areas far from urban areas are mostly served by county medical community. While suburban or urban areas closer to cities are mostly a mix of the two forms^14^.

Based on the existing practice, Tianjin's HMS follows the central government's design to build an analytical framework. We focused on this system framework, analyzed the contents of the HMS in Tianjin and develops research hypotheses.

### HMS on preference for healthcare and hypotheses

In our study, the preference for healthcare (PH) refers to an individual's preference of primary care providers based on the individual's health needs and expectations when faced with different health care options.

#### Separate treatment of emergencies and slows on PH

The “Separate treatment of emergencies and slows” emphasizes grading patients based on the type and severity of their diseases and the degree of difficulty of treatment, selecting different levels of healthcare facilities for appropriate consultation, and gradually achieving a medical process from general to specialization.

Firstly, from the supply side, policy can achieve a hierarchical order of care by establishing a well-organized division of labor. Healthcare accessibility and drug security are the basis for institutional division of labor^15^. (1) *Healthcare accessibility.* When faced with common diseases, accessibility is a primary factor that cannot be ignored^8^. Large distances limit options, with longer travel times to healthcare facilities and a lower likelihood of selecting a primary facility^16,17^. Furthermore, speed to care is critical due to the importance of daily care for common diseases, particularly when patients choose a primary facility. Accelerating the registration and treatment processes may result in a more efficient flow of patients^8,18,19^. (2) *Drug supply.* The availability and variety of drugs are also influential factors in patients' preference of care^20^. Concerns about the guaranteed availability and quality of drugs can drive patients to switch to high-level hospitals^21^. At the same time, the geographical availability of drugs can motivate patients to refer back down to the primary level, especially for patients with chronic diseases^22^. Changes in the drug catalogue during the promotion of the HMS in Tianjin have improved drug accessibility in primary medical facilities, particularly with the elimination of drug mark-ups and the establishment of a tiered system for charging for healthcare services. Thus, drug supply can effectively direct patients with common diseases from high-level hospitals to primary hospitals^3,4^.

Secondly, from the demand side, patients are guided through the establishment of a tiered medical insurance system and reasonable medical pricing. (1) *Medical insurance.* There is a strong connection between medical insurance and PH. High-level hospitals are preferred by beneficiaries of government and labor medical insurance, while low-level hospitals are preferred by beneficiaries of the cooperative medical system^23,24^. Many countries have demonstrated that medical insurance reimbursement policies play an important role in encouraging patients to use primary healthcare facilities^6,25–27^. In China, universal medical insurance has increased patients' access to care, and establishing different reimbursement rates for different levels of care can effectively guide patients' preference of care and achieve systematic triage^26^. (2) *Lower healthcare costs*. In the field of health economics, the cost of healthcare is one of the main determinants of patients' choice of healthcare provider. For primary care providers, improving cost-effectiveness not only enhances their attractiveness, but also significantly influences patients' choice preferences and their transfer behavior. Some studies have shown that cost is a key factor in patient decision-making when faced with diverse healthcare options^28–30^. At the same time, relevant studies have emphasized how price transparency and cost reductions are effective in increasing patient demand for primary healthcare services, especially during non-emergency routine care^31,32^. We expect that in the context of lower healthcare costs, patients will be more inclined to choose those primary care services that are more cost-effective. This tendency not only reflects economically rational decision-making, but may also be influenced by psychosocial factors, such as a sense of reduced financial burden and a high evaluation of value for money.

#### Two-way triage on PH

The first-contact care at the primary is mainly directed from the supply side. The main focus is on increasing investment in primary healthcare facilities and improving the availability of human resources. The main attraction for patients is the level of primary healthcare facilities and physicians' services. (1) Healthcare facilities. Since China's healthcare reform in 2009, significant investments have been made and many primary healthcare facilities have been built and upgraded^33^. Governments at all levels have invested significant resources in supporting projects such as primary healthcare infrastructure development and manpower training^34^. Studies have shown that patients are less likely to bypass local health care facilities with high technical capacity^35^. In urban areas, community health facilities (CHFs) with some diagnostic capacity (e.g., complete blood counts, liver and kidney function tests, lipid analysis, x-rays, and ultrasound) play an important priming role^36^. Thus, in the context of China's most recent healthcare reform, improving facility standards is critical and has a significant impact on improving patient experience^7,34^. (2) Physicians' services. Actually, despite the Chinese government's strong investment in grassroots level, public health services in primary facilities generally fail to gain the trust of patients^4,7^. This is closely related to the level of service provided by physicians in primary healthcare facilities. The degree of specialization, quality of service and level of reputation of medical providers have a significant impact on patient satisfaction^12^. Numerous studies have shown that those patients who choose to bypass primary care regard the standard of doctor's services and the use of past experience as important factors in their choice of consultation^4,37^. In short, having better physicians will more effectively direct the flow of patients to primary healthcare. In addition, a good family doctor system has been shown to be positively associated with better health outcomes and lower healthcare costs^38,39^. Tianjin has taken the lead in developing family doctor contracting services since the policy's implementation, and the contracting rate has reached a high level. A large body of evidence suggests that residents' level of trust in their family doctors influences their choice of primary care services^39,40^.

During the implementation of HMS, there is a lack of two-way referral channels in Tianjin, and the resources of secondary facilities are not fully utilized. Patients expect upward referral more than primary care first consultation, while upward referral from primary healthcare facilities has become the norm, referral from high-level hospitals to primary hospitals has been delayed. The establishment of referrals is entirely dependent on doctor initiative and patient cooperation, with convenience of referral and standardization of treatment being two critical factors. (1) *Referral convenience*. Convenience of referral is a key factor influencing PH. Relevant studies have shown that ease of referral process affects patient health outcomes and well-being, which in turn affects patients' health-seeking behavior^41^. As a result, effective referral systems and collaboration between upper and lower levels of care are required. (2) *Treatment Standards.* Evidence suggests that effective treatment standards can help the HMS be implemented successfully^42^. Inter-professional teams must provide standardized, continuous care based on a uniform standard of treatment. Clinical pathway integration has been shown in studies to be critical in the practice of integrating healthcare systems^43^. As a result, systematic and standardized process support is required to ensure that all patients are treated in a coordinated and effective manner^44^. Furthermore, with high-speed development in the information age, the ability to provide a standardized process for internet access is an important factor in patient healthcare-seeking behavior^45^.

#### The mediating effect of Upper and lower linkage

The analysis of the HMS contents reveals that the HMS aims to establish a healthcare service system with an orderly division of labor and mutual collaboration, as well as certain integrated functions, rather than a system with clear distinctions in terms of functions. Therefore, the degree of collaboration between the three levels of facilities, or the "Upper and lower linkage", enhancing overall service delivery rather than merely distinguishing the functional roles of each facility level^46^. A strong coordination mechanism in the HMS can improve the continuity of care for patients while effectively reducing the overall healthcare burden on society.

The creation of tightly-knit medical alliance and the sharing of healthcare resources are the most important components of the " Upper and lower linkage" mechanism. (1) *Medical alliance*. The integration of the three levels of hospitals in Tianjin's HMS practice is manifested in the formation of four different models of medical alliance, namely urban medical community, county medical community, cross-regional specialist alliances, and telemedicine collaboration networks^41,47^. We emphasize ensuring continuity of patient services through orderly collaboration across medical alliances. Under the stratified treatment strategy, patients are stratified according to the severity of their illnesses in order to prioritize the order and location of treatment, with common and frequently occurring diseases treated at primary health-care facilities, and complex or acute illnesses, such as those requiring surgery and hospitalization, treated at secondary and tertiary hospitals. This strategy directly affects patients' preferences for healthcare providers, with primary care providers being preferred because of their enhanced ability to handle common and multiple diseases. Upper and lower linkage as a mediating factor implies the optimization of resource allocation (e.g., sharing and mobility of medical personnel, technology, equipment, etc.) through medical alliance or other forms of cooperation among healthcare institutions. This linkage ensures that even when primary care providers are faced with more complex cases, they can receive support from higher hospitals through collaboration or rapid referral. In the strategy of separate treatment of emergencies and slows, the upper and lower linkage enhances the ability of primary care providers to deal with complex cases, which reduces the number of cases in which patients directly choose higher-level hospitals due to concerns about the lack of capacity of primary care providers, and thus strengthens patients' preference for primary care providers. (2) *Quality resources sharing and sinking*. The primary goal of upper and lower linkage is to increase the capacity of primary healthcare services by facilitating the vertical flow of healthcare resources and encouraging the sharing and sinking of quality healthcare resources at the primary level. To improve the standard of primary care, quality resources are shared through the dispatch of specialists, co-construction of specialties, and operational guidance. Many studies have examined the importance of sharing healthcare resources among healthcare facilities for service delivery capacity^42,48,49^, and have confirmed that collaboration among healthcare facilities is critical for improving healthcare system integration^50^.Under the two-way triage strategy, the establishment of a clear referral mechanism allows for the smooth transfer of patients between primary care and higher-level hospitals as required by their condition. This not only optimizes the process of patient treatment, but also makes primary care the preferred point of care for patients, especially in the case of common and multiple diseases. In the two-way triage system, the upper and lower linkage strengthens the service capacity of primary care providers and their ability to cope with complex conditions by ensuring the rapid flow of information and resources between primary care and higher level hospitals. This linkage reduces the need for multiple transfers due to complex conditions and improves the efficiency of primary care and patient satisfaction, thus enhancing patients' preference for primary care.

**Figure 2:**
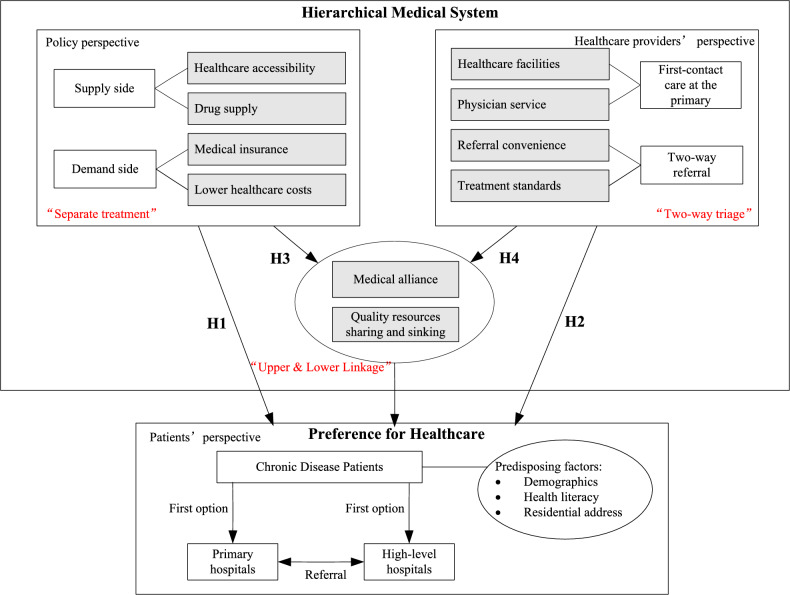
Theoretical model of HMS on Healthcare-seeking behaviors.

Based on the above analysis we present the analytical framework as shown in Fig. 2 and make the following hypothesis.

- **H1** Separate treatment of emergencies and slows has a positive impact on PH.
- **H1a** Healthcare accessibility has a positive effect on PH.
- **H1b** Drug supply has a positive effect on PH.
- **H1c** Medical insurance has a positive effect on PH.
- **H1d** Lower healthcare costs has a positive effect on PH.
- **H2** Two-way triage has a positive effect on PH.
- **H2a** Healthcare facilities has a positive effect on PH.
- **H2b** Physicians' services has a positive effect on PH.
- **H2c** Referral convenience has a positive effect on PH.
- **H2d** Treatment Standards has a positive effect on PH.
- **H3** Upper and lower linkage has a mediating effect between separate treatment of emergencies and slows and PH.
- **H4** Upper and lower linkage has a mediating effect between two-way triage and PH.

## Research design

### Research sample

**Figure 3:**
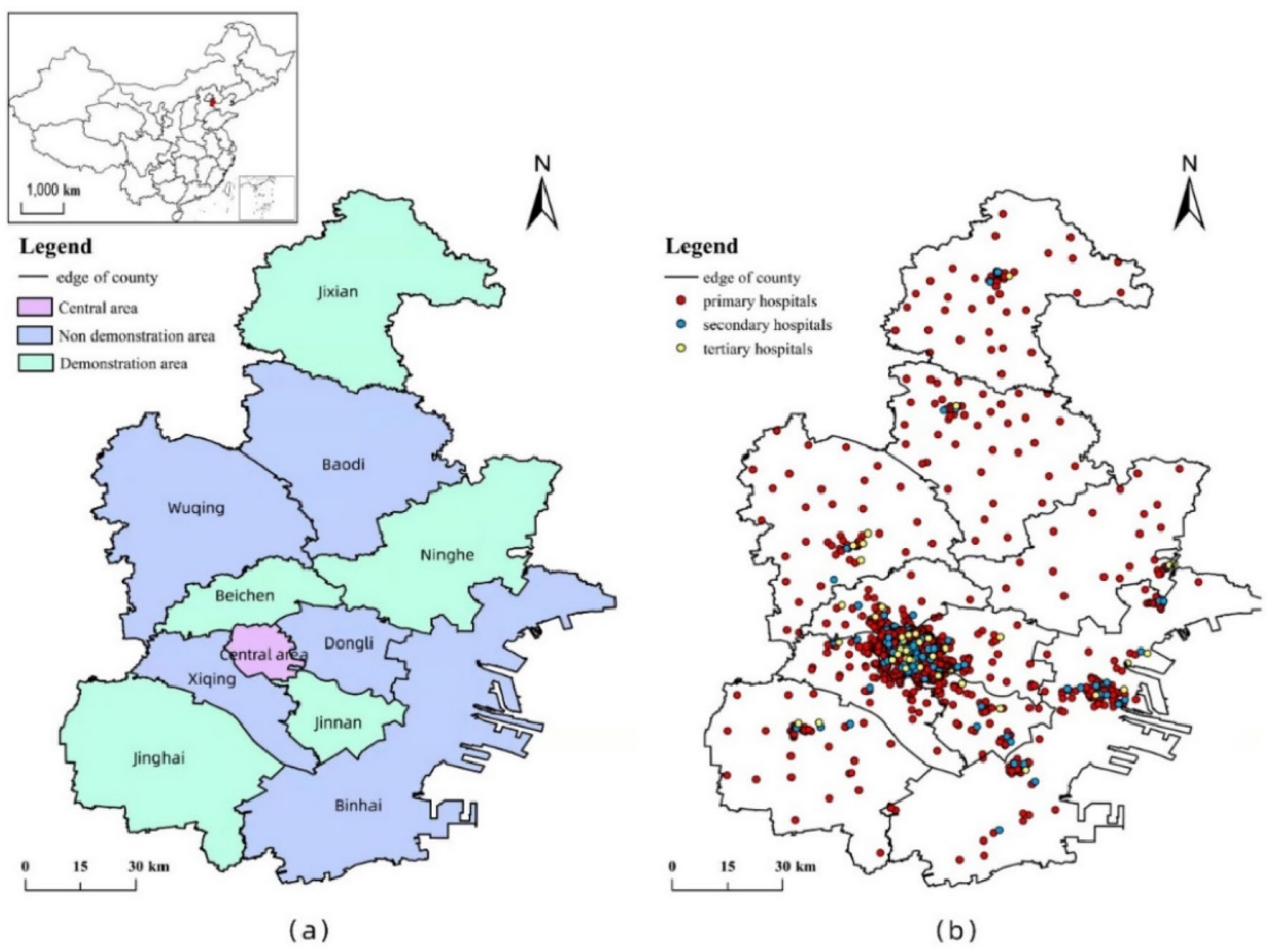
Study-area overview.

Tianjin is the largest port city in northern China, a municipality directly under the central government, a national central city and the gateway to the opening up of northern China approved by the State Council. With a total area of 11,966.45 square kilometers and a resident population of 13,866,000, Tianjin is one of the representatives of mega-cities in the north. The city has 16 districts, of which Heping, Hexi, Nankai, Hedong, Hebei and Hongqiao districts are the central areas in the city, while the remaining 10 districts belong to the surrounding areas of the city center. Since 2015, Tianjin's 16 administrative districts have been implementing the HMS to varying degrees. Among them, the five districts of Beichen, Jinan, Jinghai, Ninghe and Jizhou are national county-level medical alliances pilots, which have better practice strength in the HMS. The regional situation in Tianjin and the distribution of healthcare facilities at each level are shown in Fig. 3.

By the time of this survey, Tianjin has 45 tertiary hospitals, 76 secondary hospitals, and 655 primary hospitals, the vast majority of Tianjin's secondary and tertiary hospitals are concentrated in the six central districts. Primary hospitals continue to dominate the surrounding districts and counties. While residents of the city's six districts have greater convenience in deciding which healthcare facility to visit, residents of the suburbs are more diverse in terms of healthcare-seeking behavior due to the unequal distribution of healthcare resources. More details on the different levels of hospitals are provided in Appendix 1.

### Data collection

The data used in this study are from the Tianjin Primary Health Services Survey 2020 which is based on the Tianjin 14th Five-Year Plan conducted every five years by the Tianjin Municipal Health Commission, covering all 16 districts in Tianjin. The data collection period was from March 1 to June 1, 2020 and a total of 9,699 households and 22,877 people were polled. A sample of 12,230 patients with chronic diseases (including hypertension, diabetes mellitus, stroke and coronary heart disease) who can choose primary hospitals were selected as the sample for this study for the main objective of the HMS is to promote first-contact care at the primary for common diseases. All research was performed in accordance with relevant guidelines and consent was obtained from all participants.

Abnormal responses were removed from the questionnaires, after screening and sorting, there were 11,200 valid questionnaires which entered into the database using Epidata3.1, and check files were created at the time of entry for verification and logical error checking. The study received ethical approval from the local ethics committee of the Tianjin Municipal Health Commission. The survey did not cause any psychological or physical harm to the participants who provided verbal informed consent prior to inclusion. The social demographic features of participants are shown in Table 1. Among the 11,200 respondents who participated in the survey, the gender distribution was relatively balanced, with slightly more women than men. The age distribution shows a relatively high percentage of people aged 65 and above and those under 34, at 30.5% and 23.5% respectively, demonstrating a wide range of participation from different age groups. In terms of education level, the majority of respondents had an primary school education background, followed by high school and illiteracy. The data on areas of residence show a more balanced distribution of respondents in urban centers and demonstration and non-demonstration areas. Regarding the type of household registration, permanent residents accounted for the majority, with a share of 58.8%, reflecting the stable contribution of the permanent population to the region.

**Table 1:** Social demographic features of participants (*N* = 11,200).

Category		Options	Responses	Percentage
Gender		Male	5555	49.6
		Female	5645	50.4
Age		< = 34	2632	23.5
		35–44	1120	10
		45–54	1490	13.3
		55–64	2542	22.7
		> = 65	3416	30.5
Education		Illiteracy	2756	24.61
		Primary school	5080	45.36
		High school	2976	26.57
		Bachelor	385	3.44
		> = Master	2	0.02
Residential address	Central area/demonstration area	Heping District	585	5.22
		Hexi District	687	6.13
		Hedong District	627	5.60
		Hebei District	635	5.67
		Nankai District	590	5.27
		Hongqiao District	559	4.99
		Beichen District	717	6.40
		Jinnan District	624	5.57
		Jinghai District	674	6.02
		Ninghe District	785	7.01
		Jizhou District	821	7.33
	Non-demonstration area	Dongli District	610	5.45
		Xiqing District	760	6.79
		Binhai New Area	727	6.49
		Baodi District	840	7.50
		Wuqing District	999	8.92
Household type		Permanent residents	6586	58.80
		Temporary or foreign residents	4614	41.20
Total		11,200		

### Measuring tools

In order to give the respondents a clearer understanding of the content of the HMS and to develop a comprehensive set of indicators, we searched for and made use of a number of well-established scales such as the WHO Primary Care Assessment Tool (PCET)^51^, the ADHD Questionnaire for Primary Care Providers (AQ-PCP)^52^, the Primary Care Assessment Tool (PCAT)^53,54^. Due to the specificity of China's HMS and the fact that the majority of respondents are elderly, the questionnaire should be user-friendly, with clear and precise questions, and should take into account the specificities of policy implementation so that respondents can provide clear answers to the questions. At the start of the measurement system development, we referred to Farh's management model based on the Chinese context, and the item wording was transcribed and merged in the Chinese context to develop a scale system that is adapted to the policy context^46^. The questionnaire is based on self-completion questions and contains five main sections: " personal information ", perceived understanding of policy’s " separation of urgent and slow treatment ", " two-way triage ", " upper and lower linkage " and one’s own " preference for healthcare ". Please refer to the Appendix for details. A pre-survey was conducted at the beginning of the formal survey and the unreasonable and undifferentiated question items were removed before the formal analysis, the final retained question items are shown in Appendix 2.

### Research methods

SPSS 23.0 and AMOS 24.0 were chosen to conduct this study. Firstly, consistency coefficient test, the Kaiser–Meyer–Olkin (KMO) test, and Bartlett Test were performed, followed by correlation and regression analyses to estimate the effect of the components of “separate treatment” and “two-way triage” on PH after controlling for other factors. In the regression analysis, we used t-tests to assess whether individual variables have a significant effect on the dependent variable after controlling for other variables, and F-tests to assess whether the explanatory power of all the independent variables in the model jointly on the dependent variable is significant. Finally, structural equation modeling (SEM) was used to validate the direct effect of "separate treatment" and "two-way triage" on PH and the mediating effect of "upper and lower linkage".

### Informed consent

All research was performed in accordance with relevant guidelines and consent was obtained from all participants.

## Result analysis

### Reliability and validity test

In order to make the findings of the sample of chronic disease patients extracted from this study reliable, a validated factor analysis was used . The results of the KMO test and Bartlett test for the HMS scale in this study are shown in Table 2. The overall reliability of the questionnaire was Cronbach's ɑ = 0.879 and the validity was KMO = 0.965. Cronbach's ɑ > 0.75 and KMO > 0.8 for each scale, p < 0.01, indicating that the overall scales have good internal consistency and structural validity.

**Table 2:** Reliability and validity analysis results to the scales.

Scales	Items	Items	Score mean (SD)	Factor loading	Cronbach’s *ɑ*	KMO	AVE	C.R	Bartlett
HL	Health literacy	HL1	3.009 (1.311)	0.783	0.893	0.823	0.522	0.883	***
		HL2	3.015 (1.304)	0.802					
ST	Healthcare accessibility	HA1	3.994 (1.282)	0.800	0.808	0.804	0.563	0.795	***
		HA2	2.003 (1.459)	0.732					
	Drug supply	DS1	3.993 (0.536)	0.811					
		DS2	2.008 (1.259)	0.793					
	Medical insurance	MI1	2.000 (0.707)	0.687					
	Lower healthcare costs	HC1	4.005 (0.792)	0.700					
TT	Healthcare facilities	HF1	2.002 (0.920)	0.883	0.732	0.886	0.621	0.896	***
		HF2	4.006 (1.427)	0.847					
	Physician service	PS1	3.002 (1.203)	0.712					
		PS2	2.999 (1.360)	0.748					
		PS3	2.015 (1.131)	0.832					
		PS4	4.008 (0.543)	0.754					
	Referral convenience	RC1	3.010 (1.311)	0.901					
		RC2	3.005 (0.604)	0.923					
	Treatment standards	TS1	1.000 (1.202)	0.724					
		TS2	1.994 (1.095)	0.803					
UL	Medical alliance	MA	0.990 (0.914)	0.855	0.852	0.905	0.563	0.877	***
	Quality resources sharing and sinking	QR	4.000 (1.093)	0.838					
PH	Preferred first visit	PF	2.493 (1.120)	0.899	0.902	0.932	0.602	0.910	***
	Intention to refer	IR	2.995 (0.823)	0.912					

“***”,P < 0.01;“**”,P < 0.05; “*”, P < 0.1;

HL = Health Literacy; ST = Separate Treatment of Emergencies and Slows; TT = Two-way Triage; UL = Upper & lower Linkage; PH = Preference for Healthcare.

### Correlation analysis

To further determine the relationship between the variables, a correlation analysis was conducted on each variable and the results are shown in Table 3.

**Table 3:** Correlations between variables.

Variables	RA	AG	GE	ED	HT	HL	ST	TT	UL	PH
RA	1									
AG	− 0.014	1								
GE	0.005	− 0.001	1							
ED	0.022	− 0.213**	0.008	1						
HT	− 0.142**	− 0.112*	0.001	− 0.022	1					
HL	0.215**	− 0.298***	− 0.032	0.342***	0.001	1				
ST	− 0.134*	− 0.233**	0.022*	− 0.122*	0.213**	0.111**	1			
TT	− 0.097*	− 0.101*	0.002	− 0.232**	− 0.322***	− 0.112**	0.112**	1		
UL	− 0.221**	− 0.432***	− 0.089*	− 0.211**	− 0.112**	− 0.122**	0.213**	0.089*	1	
PH	0.398***	0.442***	0.003	0.001	− 0.303***	0.230**	0.522***	0.421***	0.349***	1

“***”,P < 0.01;“**”,P < 0.05; “*”, P < 0.1;

RA = Residential address; AG = Age; GE = Gender; ED = Education; HT = Household type; HL = Health literacy; ST = Separate treatment of emergencies and slows; TT = Two-way triage; UL = Upper & lower linkage; PH = Preference for healthcare.

Table 3 shows that the correlation coefficients between the variables are less than 0.8, and the Variance Inflation Factor (VIF) verifies that there is no multicollinearity between the variables, so the next analysis can be conducted. Age, gender, education level, household type and health literacy are all correlated with the four main variables in this study, which indicates that there is a certain degree of variation in the PH and the understanding of the HMS among different groups of people. Therefore, the use of these variables as control variables in the model helps us to eliminate confounding factors.

### Hypothesis testing of the basic model

Table 4 analyses the impact of “Separate treatment of emergencies and slows” and “Two-way triage” on the PH, controlling for demographic characteristics and level of health literacy, with a total of 16 models after progressive introduction of control variables.

**Table 4:** Regression of the basic models.

Variables	Model 1	Model 2	Model 3	Model 4	Model 5	Model 6	Model 7	Model 8
Separate treatment of emergencies and slows								
HA	0.402***							
DS		0.331***						
MI			0.430***					
HC				0.324***				
Two-way triage								
HF					0.198***			
PS						0.211***		
RC							0.334***	
TS								0.253**
Control variables								
RA								
HL								
AG								
GE								
ED								
HT								
*R*^2^	0.162	0.110	0.185	0.105	0.039	0.045	0.112	0.064
*F*	110.300***	129.412***	45.213*	34.132***	56.224**	48.521***	109.311***	42.320*
*D-W*	2.064	2.107	1.950	2.088	1.958	1.908	2.108	1.958
*N*	11,173	11,195	11,188	11,200	11,122	11,189	11,189	11,194

	Model 9	Model 10	Model 11	Model 12	Model 13	Model 14	Model 15	Model 16
Separate treatment of emergencies and slows								
HA	0.364***		0.235**	0.222**	0.187**	0.333***	0.123**	0.202*
DS	0.243***		0.211**	0.231*	0.240***	0.199**	0.310***	0.299***
MI	0.400**		0.332***	0.390***	0.301***	0.234**	0.200**	0.082
HC	0.219**		0.188**	0.104**	0.122**	0.210**	0.052	0.177**
Two-way triage								
HF		0.122**	0.109*	0.083*	0.090	0.152**	0.134**	0.062
PS		0.130**	0.111*	0.102*	0.120*	0.077*	0.128*	0.100*
RC		0.321***	0.303***	0.258**	0.222**	0.303***	0.292***	0.222**
TS		0.152**	0.122*	0.043	0.102*	0.082*	0.162**	0.055*
Control variables								
RA				0.032**				0.022
HL					0.321**			0.200**
AG						0.432***		0.302***
GE						− 0.013		− 0.009
ED						− 0.003		− 0.001
HT							− 0.190**	− 0.123**
*R*^2^	0.664	0.643	0.866	0.908	0.911	0.965	0.893	0.978
Adjust *R*^2^	0.633	0.604	0.842	0.887	0.890	0.945	0.864	0.953
*F*	21.210***	14.403**	15.212***	13.452**	9.202***	14.525***	11.345**	8.421**
*D-W*	1.965	1.987	2.012	1.984	1.908	1.960	1.907	2.109
*N*	11,167	11,156	11,155	11,144	11,156	11,127	11,122	11,104

“***”,P < 0.01;“**”,P < 0.05; “*”, P < 0.1

RA = Residential Address; AG = Age; GE = Gender; EU = Education; IN = Income; HT = Household Type; HL = Health Literacy; HA = Health accessibility; DS = Drug supply; MI = Medical insurance; HC = Health costs; HF = Health facilities; PS = Physician service; RC = Referral convenience; TS = Treatment standards.

Models 1 to 8 indicate that when the four variables in "separate treatment of emergencies and slows" (ST) and the four variables in "two-way triage" (TT) are introduced separately, both ST and TT have a positive effect on PH. Models 9 to 11 show that the findings still stand when the four variables of ST and TT are introduced at the same time. Whether the variables are introduced gradually or simultaneously, the results do not change significantly, indicating the robustness of the model. H1 and H2 were found to be valid. In order to analyze the effect of different group characteristics in more detail, we next gradually introduce control variables.

The address variable was introduced in model 12. Non-demonstration areas are assigned a value of 0, demonstration areas and central areas are assigned a value of 1. Although the inclusion of RA caused some variables to be insignificant, the overall effect of all variables on PH was significant. On the one hand, this indicates that patients in the city center are generally more willing to seek primary care and referral than those in the suburbs, on the other hand, it shows that the construction of national demonstration areas has been effective in guiding patients with chronic diseases to seek care at the primary level. Health literacy was introduced in Model 13. The positive effect of the level of health literacy can be found to be highly significant, indicating the importance of health literacy to the PH, which is in accordance with the results of most existing studies^36,37^. Age, gender and education level were introduced in Model 14. It can be seen that the effect of gender is insignificant, but age plays a positive role, with older age being more able to follow the design of the HMS. The effect of educational level is not significant, probably due to the generally low educational level of the survey sample.

A Household type was introduced in Model 15. Permanent residents are assigned a value of 1 and temporary or foreign residents are assigned a value of 2. It can be seen that permanent residents are more willing to accept the HMS, probably because most of the foreign residents are "new immigrants" in recent years, and they do not trust local primary healthcare facilities. Many foreigners come for the quality education and healthcare resources of the big cities, preferring the big hospitals in the city center and choosing the larger hospitals when they are sick.

All control variables are introduced in model 16. It can be noticed that most of the results do not change significantly, but MI and HF show insignificant. Therefore, H1a, H1b, H1d and H2b, H2c, H2d are all valid for this study.

### Hypothesis testing of the mediation model

Table 5 shows that the standardized path coefficient for the effect of ST on PH is 0.421, p < 0.01, with a significant positive effect. The standardized path coefficient for TT on PH is 0.382, p < 0.01, with a significant positive effect. The standardized path coefficient of UL on PH is 0.320, p < 0.01 which reflects UL may have a mediating effect between the two. Regression coefficients and bootstrap tests were conducted in AMOS and the model was fitted by importing the sample data with UL as the mediating variable. The fitting indices were χ2/df = 1.891; GFI = 0.943; AGFI = 0.940; NFI = 0.942; IFI = 0.933; CFI = 0.950; RMSEA = 0.022. All were within the recommended range and the first step of the mediation test was passed. Therefore, the sample data can be shown to be operational with the help of the fitting test, and the results are shown in Table 5.

**Table 5:** The bootstrap test of the mediation model.

Hypotheses	Path	Effect	Bias-corrected percentile		C.R	*p*	Decision
			Lower	Upper			
H3	ST → PH	0.421	0.344	0.531	6.784	***	Supported
	UL → PH	0.320	0.274	0.356	9.428	***	
	ST → UL → PH	0.056	0.050	0.063	8.343	***	
H4	TT → PH	0.382	0.311	0.430	7.832	***	Supported
	UL → PH	0.320	0.274	0.356	9.428	***	
	TT → UL → PH	0.042	0.038	0.047	5.604	***	

“***”,P < 0.01;“**”,P < 0.05; “*”,P < 0.1;

ST = Separate treatment of emergencies and slows; TT = Two-way triage; UL = Upper & lower linkage; PH = Preference for healthcare.

Through the structural equation modeling, it can be found that ST has an indirect effect on PH through UL, with an indirect effect is 0.056, and the bias-corrected range is 0.050 ~ 0.063, indicating the existence of indirect effect. TT has an indirect effect on PH through UL, with an effect is 0.042, and the bias-corrected range is 0.038 ~ 0.047, indicating the existence of indirect effect. Therefore, the research hypotheses H3 and H4 were validated. In addition to this, ST and TT have a direct effect on PH, with effect values of 0.421 and 0.382. Therefore, hypotheses H1 and H2 were validated.

## Discussions

This study explores how each element of the HMS (separate treatment of emergencies and slows, two-way triage, and upper and lower linkage) affects the PH of patients with chronic diseases by constructing a model with four hypotheses using data from Tianjin Primary Health Services Survey. The findings support most of the hypotheses and provide empirical evidence for understanding the actual impact of the HMS.

First, this study found that healthcare accessibility, drug supply, and lower healthcare costs significantly increased the likelihood of patients choosing primary care in the context of separate treatment of emergencies and slows. This is consistent with prior research. Previous studies have found that transportation barriers to healthcare underutilization are related, and that greater distances may deter patients or influence their choice of primary hospitals^8,16,17^. Prior studies have shown that the availability of medicines can be effective in directing referrals of patients with common illnesses from higher-level large hospitals to primary care, especially for patients with chronic conditions^20–22^. In addition, lower healthcare costs in primary care settings being a significant factor influencing patients' choice of these settings^31,32^. This highlights the importance of cost-effective healthcare in promoting utilization of the healthcare system and ensuring access to healthcare for patients with chronic diseases. Patients are very sensitive to the costs incurred in accessing healthcare and rational healthcare pricing is key to achieving triage. By analyzing data from Tianjin, this study not only verifies that these factors are equally applicable in the HMS in China, but also demonstrates how the policy can have similar effects in a wider range of regions and populations. The generalized benefits of such a policy suggest that separate treatment of emergencies and slows is not only an innovation in the healthcare system, but also a practice of social justice. It enhances public health by optimizing the allocation of resources, especially in resource-limited settings, and ensures that all types of patients have access to necessary medical support, reducing social inequalities in healthcare delivery. These findings provide important empirical support for policymakers, emphasizing that when promoting the strategy of separate treatment of emergencies and slows the potential impact on different populations should be considered comprehensively to ensure wide acceptance of the policy and maximize social benefits. Although most of the hypotheses were as expected, the study found no significant effect of medical insurance on patients' choice of a primary care provider, which may be related to the regional characteristics of the sample and the comprehensiveness of health care policies, suggesting that in a full-coverage health insurance system, the effect of insurance on choice may be offset by other factors. While previous studies have found health insurance to be a major driver of patient choice, the results of this study provide a different perspective, suggesting that this influence may vary across populations and policy environments^6,25–27^. For example, one piece of evidence from Jiangsu Province suggests that increased reimbursement does not affect diabetic patients' choices among different providers^55^, in addition to findings from a study of internal migrants suggesting that a universal health insurance scheme improved healthcare access for older Chinese people, but played little role in categorizing patients and guiding healthcare behaviors^56^.

Secondly, in exploring the two-way triage effect, our findings show that the Physicians' services, as well as the referral convenience and the Treatment Standards, all positively influence the choice of patients' consultation. The empirical analysis in this study reveals that improving the service quality of medical institutions and standardizing the diagnosis and treatment process not only enhances patients' trust in medical services, but also deepens their reliance on primary care. This finding is consistent with previous research findings that suggest that good organizational structure and processes can increase user satisfaction and dependence^39–43^. It is worth noting that although the physical condition of healthcare facilities and the modernization of equipment had an effect on patient attraction, this effect was not significant in this study. This may indicate that with the popularization of basic healthcare facilities and the increasing demand of patients for quality of healthcare services, relying solely on the improvement of facilities is no longer sufficient as a major factor in attracting patients. Instead, the quality of service, professionalism of the medical process, and personalized healthcare experience have become more important considerations for patients when choosing where to seek care. Overall, these findings underscore the need for improved service quality and process standardization in health system policies, as well as the importance of creating an efficient and trusting healthcare environment to promote patient loyalty and trust in primary care.

Different groups have various responses to the understanding of the HMS, such as differences in health literacy level, age and type of household. Patients with higher levels of health literacy showed a stronger willingness to seek primary care and to accept two-way referrals. The older patients with chronic diseases were more willing to seek primary care and to cooperate with referrals. For permanent ill residents it is more acceptable to seek first-contact care at the primary. In addition, we found that the foreign residents, who are more health awareness, are less likely to understand the HMS and tend to go to high-level hospitals when they have common chronic diseases and are less inclined to refer. Therefore, it is particularly important to improve the service capacity of medical institutions.

Finally, the mediating role of the upper and lower linkage is particularly evident in HMS. This study confirms this for the first time. This suggests that for policies and the service capacity of healthcare institutions to be most effective, they must be achieved through cooperation and resource sharing between upper and lower levels of the organization. This follows the findings of previous studies and provides further evidence of the critical role of the upper and lower linkage mechanism^42,48,49^. We speculate that, depending on the research perspective and analytical approach, the upper and lower linkage may have both mediating and regulating roles. This is a worthy direction for future research. In HMS, the core is not just the division of labor, but how to achieve more effective cooperation through a more detailed division of labor.

According to the findings of this study, the importance of "Upper and lower linkage" should be emphasized. Efforts should be made to guide the system and improve the service capacity, while also emphasizing healthcare facilities cooperation. As a result, the formation of tightly-knit medical alliance of various levels and categories should continue to be promoted, with a division of labor, led by tertiary hospitals or hospitals with greater operational capacity, depending on local conditions. Ensuring the service qualifications of primary healthcare facilities is a necessary foundation for the implementation of the HMS. Therefore, we should promote the sharing and sinking of quality healthcare resources and enhance the standard of service through the dispatch of specialists, joint construction of specialties and business guidance. The contracted family doctor service has been deepened within the tightly-knit medical alliance. The contractual service relationship with community residents is based on a certain payment mechanism, and the contracted residents are provided with basic medical services according to the contract. In addition, the establishment of a system of the HMS requires the understanding of residents and the support of society.it is necessary to raise residents' literacy of the HMS and change their perception of the primary healthcare structure. This requires the establishment of a notification system of the HMS and the strengthening of the linkage between the upper and lower levels in the medical alliance, the convenience of access to health care, health insurance benefits and graded services.

The study has two main contributions: (1) Based on the Andersen service model, this study proposes a theoretical framework for the impact of the HMS on healthcare-seeking behaviors, using the existing HMS of the Chinese government as a framework. The study investigates the impact of the HMS as an external influence on the healthcare preference of patients with chronic diseases using individual factors as control variables, enriches the theoretical perspective of the HMS. (2) Taking a large city representative of reform practice in northern China as the research object and chronic disease patients as the sample, the study focuses on exploring the mechanism of the effect of the practice of the HMS on the preference of chronic disease patients for healthcare, which enriches the research on the HMS and provides a reference for the government to promote the HMS in the future.

Our study has several limitations as follows: Firstly, this study is based on self-administered questionnaire data on the effects of the HMS. Although it can reflect patients' perceptions of the HMS and its impact on the preference for healthcare, the data are the subjective opinions of the patients and cannot objectively reflect the actual impact of the policy measures. Secondly, the survey phase was in a state of blockade in most parts of China, in which case residents may prefer to choose the nearest primary health care center in the absence of a critical emergency. This situation may have had an impact on the results. Third, the theoretical framework of this study is based on the framework of the HMS proposed by the Chinese government, which in actual fact contains a large number of variables. In order to make the survey easier to conduct, we have simplified the variables and question items, and the questions are unavoidably subjective. Finally, all the data for this study came from the city of Tianjin. Although it can reflect the situation of the HMS in Tianjin, the level of the HMS varies by region and city and cannot fully reflect the national level, which may limit the generalizability of our findings. We will broaden the scope of the sample and increase the number of city comparisons in the future study.

## Conclusions

Based on the "management paradigm of the Chinese context", this study constructs a model based on the framework and practical experience of the HMS, investigating the mechanism of influence of chronic disease patients' perceptions of the HMS on their PH under the framework of the system design. The study was conducted with the help of the proposed hypothesis for data validation and fulfilled the expected objectives of the study, providing an empirical reference for further evaluation and investigation of the practice of the HMS in China.

## Data availability 

All data generated or analysed during this study are included in this published article [and its supplementary information files.

### Supplementary Information

Supplementary Information.
