# Supplementary material for: Healthcare preferences of chronic disease patients under China's hierarchical medical system: an empirical study of Tianjin's reform practice
Source: Sci Rep. 2024 May 21;14:11631. doi: 10.1038/s41598-024-62118-8 (PMC11109171; doi:10.1038/s41598-024-62118-8)
Supplement: Supplementary file 1 — Supplementary Information. [file 41598_2024_62118_MOESM1_ESM.docx]

Appendix 1

**Appendix table1** Key indicators for primary/secondary/tertiary hospitals in 2020

| Indicators | Tertiary hospitals | Secondary hospitals | Primary hospitals |
| --- | --- | --- | --- |
| Number of inpatient visits | 1050562 | 180091 | 15948 |
| Number of outpatient/emergency visits | 31382952 | 11500380 | 38588176 |
| Number of beds | 36912 | 14477 | 7899 |
| Number of medical staff | 31579 | 15140 | 49236 |
| Total bed days | 9603450 | 2370000 | 234789 |
| Average inpatient cost | 22000.8 | 18027.6 | 3586.9 |
| Average outpatient cost | 488.8 | 362.3 | 182.4 |

Appendix 2

The questionnaire is based on self-completion questions and contains five main sections: " Personal information ", perceived understanding of policy’s " separation of urgent and slow treatment ", " two-way triage ", " upper and lower linkage " and one’s own " preference for healthcare ".

**Personal information.** This section includes two main sections on the patient's social demographic characteristics and health literacy. The social demographic characteristics include address, age, gender, education level and type of household registration. The address is assigned according to whether it is a central area, a demonstration area or a non-demonstration area.

**Separate treatment of emergencies and slows.** This section measures patients' perceptions of the design of the separate treatment of emergencies and slows system, both on the supply side and on the demand side. The supply side is measured in terms of healthcare accessibility and drug supply. Healthcare accessibility is determined by the distance and time of consultation. Drug supply is measured in terms of drug security and drug accessibility. The demand side is measured by the medical insurance reimbursement diversion and the reasonableness of healthcare costs.

**Two-way triage.** This section contains two main components: first-contact care at the primary and two-way referral. First-contact care at the primary covers two main areas: the level of healthcare facilities and physicians' services. The healthcare facilities are evaluated in terms of patients' satisfaction with the service capacity and environmental conditions. Physicians' services are assessed in terms of attitude, conscientiousness, patient recognition and trust. Two-way referral is measured in terms of the convenience of referrals and the treatment standards for chronic diseases. The referral convenience is measured in terms of patients' degree of understanding of internet referrals and the convenience of the referral process. The treatment standards are based on the degree of satisfaction with the rationality of the treatment system and the green channel for referrals.

**Upper and lower linkage.** The main form of linkage between different levels of healthcare institutions in Tianjin is the formation of medical alliance., quality resources from high-level hospitals can be sunk to provide assistance to primary healthcare facilities in the medical alliance. This section measures patients' perceptions of medical alliance and the degree of knowledge of resource sinking.

**Preference for healthcare.** This section contains questions about patients' willingness to seek 19-contact care at the primary and referral.

**Appendix table2** Scale items

| **Scales** | **Dimensions** | **Items** |
| --- | --- | --- |
| ***Personal information*** | Social demographic characteristics | Residential Address |
|  |  | Age |
|  |  | Gender |
|  |  | Education |
|  |  | Household type |
|  | Health literacy | I can accurately identify the symptoms of my disease. |
|  |  | I can correctly determine the severity of my disease. |
| ***Separate treatment of emergencies and slows*** | Healthcare accessibility | I can get to the nearest healthcare facility in a relatively short distance. |
|  |  | My consultation process takes less time. |
|  | Drug supply | My primary healthcare facility is more convenient for purchasing and collecting medicines. |
|  |  | My primary healthcare facility has adequate drug security. |
|  | Medical insurance | The difference in reimbursement rates is an important factor in my choice of treatment. |
|  | Healthcare costs | The cost of healthcare at my nearest primary facility is reasonable. |
| ***Two-way triage*** | Healthcare facilities | What do you think of the service capacity in primary hospitals? |
|  |  | What do you think of the environment in primary hospitals? |
|  | Physician service | What do you think of the attitude of the primary hospitals? |
|  |  | How conscientious do you think the doctors are during the treatment process? |
|  |  | How do you think the doctors are competent in their practice? |
|  |  | How much do you trust your doctor? |
|  | Referral convenience | How well do you think high-level hospitals and primary hospitals collaborate in the referral process? |
|  |  | How well do you think information is shared during the referral process? |
|  | Treatment standards | How reasonable do you think the two-way referral process is? |
|  |  | How would you rate the current green channel system in the two-way referral process? |
| ***Upper and lower linkage*** | Medical alliance | How well do you know about the medical alliance? |
|  | Quality resources sharing and sinking | How much do you know about specialists resources sharing and sinking? |
| ***Preference for healthcare*** | Preferred first visit | For common illnesses, I am willing to go to primary hospitals. |
|  | Intention to refer | I am willing to cooperate with large hospitals in making referrals to low-level hospitals. |

Notes：Excluding the basic information, all the questionnaires in this study use Likert’s 5-point method.

Patients were asked to score 1-5 on the question items in the Separate treatment of emergencies and slows scale, the Health literacy and Preference for healthcare scale, which 1 = “completely disagree”, 3 = “neutral”, 5 = “completely agree”; in Two-way triage scale, 1 = “completely unsatisfied”, 3 = “neutral”, 5 = “completely satisfied”; in Upper & lower linkage scale, 1 = “completely inconsistent”, 3 = “neutral”, 5 = “completely consistent”.
